# Mechanistic Insights into the Efficacy of Sodium Bicarbonate Supplementation to Improve Athletic Performance

**DOI:** 10.1186/s40798-016-0065-9

**Published:** 2016-10-11

**Authors:** Jason C. Siegler, Paul W. M. Marshall, David Bishop, Greg Shaw, Simon Green

**Affiliations:** 1 School of Science and Health, Sport and Exercise Science, Western Sydney University, Locked Bag 1792, Penrith, NSW Australia; 2 School of Medicine, Western Sydney University, Sydney, Australia; 3 Institute of Sport, Exercise and Active Living (ISEAL), Victoria University, Melbourne, Australia; 4 Australian Institute of Sport, Canberra, Australia

## Abstract

A large proportion of empirical research and reviews investigating the ergogenic potential of sodium bicarbonate (NaHCO_3_) supplementation have focused predominately on performance outcomes and only speculate about underlying mechanisms responsible for any benefit. The aim of this review was to critically evaluate the influence of NaHCO_3_ supplementation on mechanisms associated with skeletal muscle fatigue as it translates directly to exercise performance. Mechanistic links between skeletal muscle fatigue, proton accumulation (or metabolic acidosis) and NaHCO_3_ supplementation have been identified to provide a more targeted, evidence-based approach to direct future research, as well as provide practitioners with a contemporary perspective on the potential applications and limitations of this supplement. The mechanisms identified have been broadly categorised under the sections ‘Whole-body Metabolism’, ‘Muscle Physiology’ and ‘Motor Pathways’, and when possible, the performance outcomes of these studies contextualized within an integrative framework of whole-body exercise where other factors such as task demand (e.g. large vs. small muscle groups), cardio-pulmonary and neural control mechanisms may outweigh any localised influence of NaHCO_3_. Finally, the ‘Performance Applications’ section provides further interpretation for the practitioner founded on the mechanistic evidence provided in this review and other relevant, applied NaHCO_3_ performance-related studies.

## Key Points

- NaHCO_3_ has wide-ranging effects on mechanisms related to whole-body metabolism, specifically the [PCr]/[Pi]-power relationship, glycolytic intermediates and the intra- and extracellular distribution of metabolites and other strong ions.
- NaHCO_3_ may affect muscle physiology and motor pathways associated with early rate of force development and/or the metabolic properties associated with contractile shortening velocity, and this effect appears more pronounced in fast-twitch fibres.
- Appropriately identifying whether NaHCO_3_ ingestion may influence the underlying mechanisms associated with whole-body metabolism, muscle physiology and/or motor pathways within the context of a particular athletic endeavour or training stimulus is an important initial step when considering the use of this supplement.

## Review

### Background

**Fig. 1:**
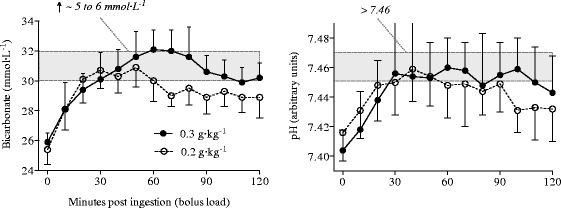
Resting changes in blood bicarbonate [HCO_3_^−^] and pH after ingesting 0.2 and 0.3 g·kg^−1^ of sodium bicarbonate (NaHCO_3_) (reproduced from Siegler et al. [3] with permission)

For over 40 years, researchers have explored the efficacy of inducing alkalosis to enhance athletic performance. Although many buffers have been studied (e.g. sodium citrate, sodium phosphate, sodium lactate), evidence supports sodium bicarbonate (NaHCO_3_) as the most consistently effective agent for improving exercise performance. Meta-analyses have reported that supplementation can result in an approximately 2 to 3 % improvement in a variety of performance measurements (e.g. power, speed, work capacity, time to failure) during both single and repeated bouts of high-intensity exercise [1, 2]. Oral administration of NaHCO_3_ generally increases blood buffering capacity (Fig. 1) [3] and is believed to attenuate the increase in intramuscular acidity synonymous with high-intensity exercise and skeletal muscle fatigue [1, 4], although the physiologic mechanisms directly responsible for performance augmentation in humans are unclear. Indeed, a large proportion of empirical research and reviews investigating the ergogenic potential of NaHCO_3_ focus predominately on performance outcomes (e.g. time to task failure, cumulative work accomplished, time trial performance) [1, 4, 5], and only speculate about underlying mechanisms responsible for attenuating skeletal muscle fatigue.

Mechanistic links between NaHCO_3_ supplementation, skeletal muscle fatigue and athletic performance are often underpinned by the belief that metabolic acidosis, or a decline in muscle pH, contributes to skeletal muscle fatigue through inhibition of various metabolic processes and/or rates of contractile cycling [6–9]. However, it has also been argued that acidosis has little influence on maximal force production [10], and in some instances may even facilitate maximal force and velocity of shortening [11]. The debate as to whether or not metabolic acidosis, or indeed the intra- and extracellular distribution of other relevant ions (e.g. Ca^2+^, K^+^, Cl^−^), has an inhibiting or facilitating effect on contractile function continues today and has been summarised in recent reviews [12–14]. The primary aim of this review was to fundamentally examine the influence of NaHCO_3_ supplementation on metabolic acidosis and mechanisms associated with skeletal muscle fatigue and athletic performance. Secondly, the mechanistic evidence presented in this review has been contextualised in the ‘Performance Applications’ section to provide contemporary, evidence-based recommendations for practitioners and athletes.

After the ‘Historical Overview’ section, studies have been broadly categorised under sub-headings, and when possible, the performance outcomes of these studies contextualized within an integrative framework of whole-body exercise where other factors such as task demand (e.g. large vs. small muscle groups), cardio-pulmonary and neural control mechanisms may outweigh any localised influence of NaHCO_3_. In many of these studies, NaHCO_3_ is also used in conjunction with an acid (e.g. ammonium chloride (NH_4_Cl)) to determine the wider contribution of acid-base perturbation on skeletal muscle fatigue; however, emphasis has been placed on reviewing only the alkalosis condition in most instances. This review does not address all performance-based applications of NaHCO_3_ supplementation in the literature, such as team sport performance [15–17], skill sports [18–20], hydration [21, 22] or the efficacy of NaHCO_3_ under hypoxic conditions [23–25].

### Historical Overview

Associations between NaHCO_3_, skeletal muscle fatigue and exercise performance can be traced back to what is believed to be the first paper published on the topic in 1931 from the Harvard Fatigue Laboratory [26]. This study was one of many in this era more broadly focused on respiratory and metabolic buffering mechanisms associated with what was termed ‘lactic acidosis’ [26]. Indeed, there was considerable interest in lactic acidosis and fatigue during muscle contractions and exercise as early as the 1900s and continuing through to the 1970s [27]. However, beyond a few subsequent investigations [28–31], research on the ergogenic potential of NaHCO_3_ did not become prominent until the mid-1970s.

The first series of exercise performance studies to be formally presented from one laboratory were from Jones and colleagues starting in 1977 [32–35]. Collectively, these were also the first studies on humans to incorporate both an alkali (NaHCO_3_) and an acid (NH_4_Cl) to explore the impact of altered acid-base status on markers of cardiovascular, respiratory and metabolic function in conjunction with exercise performance. In the first two studies, cycling at 95 % of VO_2_max to volitional exhaustion after NaHCO_3_ ingestion elicited between a 30 and 40 % performance improvement, respectively [32, 33], whereas a following study requiring a 30-s maximal effort at 100 rpm only resulted in a small but non-significant improvement in maximal power [34]. In the fourth study, incorporating an incremental cycling protocol (~16 W min^−1^ at 60 rpm to volitional exhaustion), only the NH_4_Cl condition induced a performance decrement whereas there was no difference between the NaHCO_3_ and control conditions [35]. The authors accounted for the performance discrepancies by relating exercise task and duration to a decline in muscle pH and the concurrent effect on the rate of glycolysis. Furthermore, they suggested that the beneficial effects of enhanced H^+^ removal would be more advantageous during the prolonged cycling tasks (e.g. >4 min) than during the shorter, 30-s efforts [34]. The discrepancy in performance outcomes in these studies is also the first of many examples where different exercise task demands and NaHCO_3_ dosing protocols may have impacted upon performance outcomes.

From the mid-1980s, performance-related NaHCO_3_ studies began to emerge prominently in the literature. The increased interest may have been influenced by earlier findings [32, 33], but was probably also related to the ease of oral NaHCO_3_ administration and the short time course required to acutely induce metabolic alkalosis that provided the opportunity to conduct a wide variety of performance-related studies within a relatively short period of time [36–40]. Collectively, the studies during this period that reported an ergogenic benefit of NaHCO_3_ ingestion generally adhered to a maximal or supramaximal (e.g. 125 % VO_2_max) exercise task lasting between 1 and 7 min (Table 1) [41, 42]. Many of the performance-based studies in this era also proposed a similar study rationale; one based on previous equivocal performance outcomes and/or on the attenuation of the decline in muscle pH proposed to occur during high rates of glycolytic flux after NaHCO_3_ ingestion [39, 40, 43–45]. Indeed, these rationales persist in many published papers today [46–49], further illustrating that beyond the effect on glycolysis, the other potential physiologic mechanisms responsible for performance augmentation after NaHCO_3_ supplementation have received limited attention in the applied research domain.

**Table 1:** A summary of studies conducted in the mid 1980’s that reported an ergogenic effect of 0.3 g kg^−1^ sodium bicarbonate (NaHCO_3_) supplementation on performance tasks lasting approximately −7 min

Study	Subject characteristics	Task	Control	NaHCO_3_	Improvement (%)
Wilkes et al. ‘83 [36]	University track athletes	800-m foot race	2:05.8 ± 2.1 (min:s)	2:02.9 ± 1.9 (min:s)	~2 %
Costill et al. ‘84 [40]	VO_2_max: 4.8 L min^−1^ (3.12–6.33)	5, 1-min cycling bouts (125 % VO_2_max; 5th bout to exhaustion)	113.5 ± 12.4 s	160.8 ± 19.1 s	~42 %
McKenzie et al. ‘86 [38]	VO_2_max: 3.83 ± 0.61 L min^−1^	6, 60-s cycling bouts (125 % VO_2_max; 6th bout to exhaustion)	74.7 ± 17.6 s	106 ± 6.9 s	~30 %
Gao et al. ‘86 [37]	Well-trained college swimmers (VO_2_max: 4.3 ± 0.1 L min^−1^)	5, 100-yd freestyle swim	4th bout: ~1.65 m s^−1^ 5th bout: ~1.64 m s^−1^	4th bout: ~1.62 m s^−1^ 5th bout: ~1.61 m s^−1^	~2 % ~2 %
Goldfinch et al. ‘88 [39]	Male athletes	400-m foot race	58.46 ± 2.49 s	56.94 ± 2.25 s	~3 %

Subject characteristics are represented to the level of detail provided in the original published studies. All values in the (%) improvement column were significantly different from control (*p* < 0.05)

### Whole-Body Metabolism

#### Anaerobic/Aerobic Capacity

From an energetic perspective, any physiological effects of NaHCO_3_ supplementation that translate to an increase in performance should be observed in the rates and amounts of energy provision and/or the efficiency of energy transduction to power and work. However, very little research has investigated, or indeed attempted to quantify, anaerobic and aerobic energy yields, accumulated oxygen deficits or energetic efficiency during conditions of metabolic alkalosis. Beyond a handful of studies that have modelled whole-body VO_2_ kinetics (e.g. VO_2_ fast and slow components [50–52]), most of the research on NaHCO_3_ and metabolism has been conducted on single muscle or isolated muscle groups and their metabolic response to various task demands.

From studies conducted in the 1970s [32, 53–55], a causal link between increased intramuscular lactate (La^−^) concentrations and La^−^ efflux, reduced proton (H^+^) accumulation and improved muscular performance had been posited to explain the performance improvements observed following NaHCO_3_ supplementation [6]. To further clarify the influence of NaHCO_3_ on intramuscular [La^−^] and La^−^ efflux during intense exercise, Spriet and colleagues minimised the limitations associated with in vivo contracting muscle measurement (e.g. varying work rates and metabolite accumulation) by applying continuous supramaximal stimulation (5 V at 0.5 Hz) for 20 min to a perfused gastrocnemius-plantaris-soleus (GPS) muscle group [56]. NaHCO_3_ increased the rate of La^−^ efflux from the muscle group, but did not attenuate the decline in muscle tension as compared to control conditions [56]. Methodological limitations inherent to the perfusion and stimulation protocol only allowed the authors to speculate as to whether or not the increased La^−^ efflux was a result of increased rates of glycogenolysis and/or glycolysis, or due to an increased rate of La^−^ release from the muscle.

Nearly 15 years later, Hollidge-Horvat and colleagues used human muscle tissue samples obtained at different steady-state exercise intensities (i.e. 30, 60 and 75 % VO_2_max) to investigate the mechanisms responsible for the increased La^−^ efflux by then commonly observed after NaHCO_3_ ingestion [57]. These authors eloquently detailed a complex, intensity-dependent response in key metabolic enzymes and regulatory steps after NaHCO_3_ ingestion as compared to control conditions. Briefly, the authors observed a similar increase in La^−^ efflux to that of previous studies after NaHCO_3_ ingestion, but only at 75 % VO_2_max [57]. The authors speculated that the increased [La^−^] observed in circulation may have been due to a decrease in La^−^ uptake by inactive tissue [58] or an altered H^+^ gradient on the transporters (later confirmed by others as an increase in monocarboxylate transporter (MCT) activity [59]). The authors also documented an increased degradation of muscle glycogen in the NaHCO_3_ compared to the control trials [57]. As performance was not assessed in this study, whether or not this increased reliance on muscle glycogen stores in the NaHCO_3_ trial would have influenced performance at the higher intensities remains unknown.

As the capacity to study exercising muscle metabolism in vivo improved beyond the limitations of the biopsy, others have subsequently expanded upon these findings to include exercise performance measures. Using phosphorus-31 magnetic resonance spectroscopy (^31^P-MRS) and a constant-rate progressive wrist-flexion exercise (1-s contraction/1-s relaxation) to volitional exhaustion, Raymer and colleagues reported an approximate 12 % increase in time to failure after NaHCO_3_ supplementation and attributed the performance effect to a delayed onset of intracellular acidosis (evident at ~60 % peak power) in the alkalotic condition [60]. Furthermore, concomitant to the delayed intracellular acidosis was a delay in the slow-to-rapid increase in the [PCr]/[Pi] to power output relationship (repeatedly observed in subsequent studies by this same group [61, 62]). As metabolic perturbation caused by acidosis had been associated with the inhibition of glycolysis and glycogenolysis [63], increased phosphocreatine (PCr) degradation and inorganic phosphate (P_i_) accumulation ([PCr]/[P_i_]) within the myocyte [64], the authors speculated that the delayed accumulation of intracellular H^+^ induced by NaHCO_3_ was the primary cause of the performance effect.

**Fig. 2:**
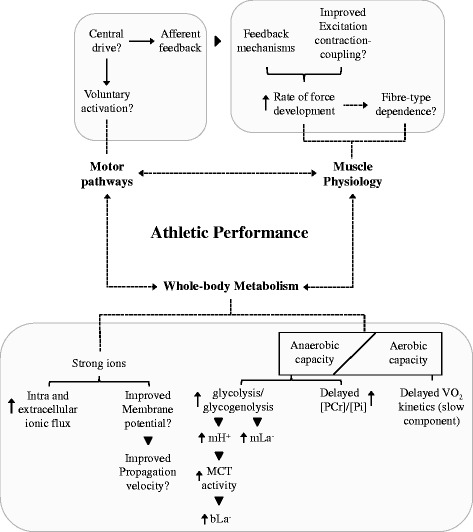
Overview of the mechanisms associated with sodium bicarbonate supplementation (NaHCO_3_) and whole-body metabolism, muscle physiology and motor pathways. Definitions for the following abbreviations are provided for mH^+^ (muscle protons), mLa^−^ (muscle lactate), MCT (monocarboxylate transporters), bLa^−^ (blood lactate) and [PCr]/[P_i_] (ratio of phosphocreatine to inorganic phosphate)

To briefly summarise, much of the research in this area has been centred around muscle metabolism, from the earlier observations on intramuscular [La^−^] and La^−^ efflux to the more recent focus on glycolytic intermediates and high-energy phosphate kinetics (e.g. PCr). NaHCO_3_ appears to effect the [PCr]/[Pi]-power relationship, glycolytic intermediates and the intra- and extracellular distribution of metabolites and other strong ions (Fig. 2) [56, 57, 60, 65, 66]. Not surprisingly, this effect is more pronounced during periods of prolonged, intense contractile activity where a larger proportion of energy provision is derived from classically defined *anaerobic pathways*. Contextually, this effect may also partially explain the ergogenic findings of many performance-based studies on athletes that have imposed a high demand on these pathways either with single or repeated bouts of intense exercise [39, 67–70].

#### Strong Ions

Inducing a state of alkalosis also affects the intra- and extracellular balance of other strong ions such as Na^+^, K^+^ and Cl^−^ [60], all of which contribute to the maintenance of skeletal muscle contractile function [13]. In general, and irrespective of acid-base balance, fatiguing exercise induces ion concentration changes between the intracellular, interstitial and extracellular compartments. Although ultimately dependent upon the level of contractile activity, as well as diffusion and perfusion limitations, muscle K^+^ will tend to decrease whereas muscle Na^+^, Cl^−^ and Ca^2+^ will tend to increase with intense exercise (H^+^ will increase in both intra- and extracellular compartments) [13]. Collectively, changes in these ionic concentrations have been demonstrated to decrease resting membrane potential and impair sarcolemmal excitability [13]. The effect of NaHCO_3_ specifically on the distribution of these ions between compartments, in particular the NaHCO_3_^−^-induced reduction in K^+^ efflux from the muscle, has been the focus of other studies investigating the effect of alkalosis on the interstitial and plasma concentrations of strong ions and skeletal muscle fatigue [65, 66, 71].

In terms of exercise performance, however, only Sostaric and colleagues have demonstrated an improvement after NaHCO_3_ supplementation [65]. By incorporating a small muscle mass, and therefore inducing a larger reduction in muscle K^+^ than commonly observed during whole-body exercise [72], the authors hypothesised that NaHCO_3_ would attenuate the augmented K^+^ efflux from the small muscle mass and therefore better preserve muscle function [65]. Subsequently, they observed an increase in total exercise time of greater than 2 min (~25 % improvement) compared to a placebo during a constant rate, progressive concentric forearm flexion exercise task to volitional exhaustion [65]. NaHCO_3_ also improved the regulation of circulating K^+^ and handling of Cl^−^ (but not Na^+^), which the authors proposed to have better preserved membrane excitability and therefore contributed to the performance improvement [65].

NaHCO_3_ appears to delay the rise in muscle interstitial K^+^ that occurs during intense contractile activity and, via this effect, may attenuate the decline in muscle membrane excitability and the extent of K^+^-induced inactivation of myocytes during such activity [11, 66]. As previously stated, this effect may have contributed to not only the performance improvement observed by Sostaric and colleagues but also the earlier findings of Raymer as both used a similar exercise performance task [60, 65]. However, these studies also illustrate the relevance of muscle mass and task demand when determining the efficacy of NaHCO_3_ as an ergogenic supplement. Issues such as agonist/antagonist muscle actions, synergistic contraction of larger muscle groups and recruitment order (e.g. Hennemen’s size principle) [73] during whole-body exercise may mitigate the performance effects observed in smaller, isolated muscles. As an example, the trend of NaHCO_3_ improving incremental exercise to volitional exhaustion in smaller muscle groups [60, 65] is less consistent when large muscle groups (e.g. cycling) or whole-body exercise is incorporated within a similar task [32, 33, 35, 74, 75] (Table 2).

**Table 2:** A summary of the results of studies investigating the influence of 0.3 g kg^−1^ sodium bicarbonate (NaHCO_3_) supplementation on whole-body and isolated muscle incremental exercise to volitional exhaustion

Study	Whole-body or isolated muscle	Incremental task	Performance effect
Raymer et al. ‘04 [60]	Isolated (forearm)	1:1 contraction-relaxation cycle; initial resistance 1.0 kg then increased 0.22 kg min^−1^	~12 % improvement
Sostaric et al. ‘05 [65]	Isolated (concentric finger flexion)	30 rep min^−1^; initial resistance 2.6 ± 0.22 W then increased 0.17 W min^−1^	~25 % improvement
Poulus et al. ‘74 [74]	Whole-body (cycling)	Fixed cadence with 10 W min^−1^ increased until volitional exhaustion)	No effect
Jones et al. ‘77 [32]	Whole-body (cycling)	20 min at power output ~33 % VO_2_max, then 20 min at 66 % VO_2_max, then 95 % VO_2_max to volitional exhaustion	~40 % improvement
Sutton et al. ‘81 [33]	Whole-body (cycling)	20 min at power output ~33 % VO_2_max, then 20 min at 66 % VO_2_max, then 95 % VO_2_max to volitional exhaustion	~30 % improvement
Kowalchuk et al. ‘84 [35]	Whole-body (cycling)	Increased 16 W min^−1^ at 60 rpm	No effect
Housh et al. ‘91 [75]	Whole-body (cycling)	Continuous (2-min stages) physical work capacity test (PWC_FT_) starting at 60 W to volitional exhaustion	No effect

All values represented as having an improvement (%) were significantly different from control or placebo (*p* < 0.05)

### Muscle Physiology

#### Influence of Fibre-Type

Although earlier work had illustrated a relationship between muscle pH and skeletal muscle force and/or muscle shortening velocity [76–78], the first to show an independent effect of acidosis on fast- and slow-twitch fibres appears to be Metzger and Moss in 1987 [79]. Using single fibres of rat soleus (SOL) and vastus lateralis (VL) muscles, previously characterised as predominately type I (SOL) or IIa and b (VL), respectively [80, 81], maximal isometric tension and maximum shortening velocity was assessed as a function of pH. Although initial declines in pH induced a concomitant reduction in tension and shortening velocity in both SOL and VL, as pH decreased below 6.2, the relative loss in tension was greater in VL [79]. The first to study the effect of NaHCO_3_ on fibre-specific force production was Lindinger and colleagues in 1990 [82]. These authors examined the effects of NaHCO_3_ on SOL and white gastrocnemius (WG) muscle tension at rest and during 5 min of intense stimulation [82]. Although NaHCO_3_ differentially altered the ionic composition (specifically K^+^, Cl^−^ and Mg^2+^) and the rate of La^−^ efflux from the muscle fibres, it had no effect on muscle tension in either muscle [82].

In 2007, Broch-Lips and colleagues stimulated the SOL and extensor digitorum longus (EDL), another muscle characterised as predominately type IIa, under normal or alkalotic conditions. In addition to the isometric force measurement, the authors also estimated rate of force development (RFD) as the maximal numerical slope value of the force development during contraction [71]. Although NaHCO_3_ induced increases in pH from 7.4 to 7.6, neither maximum force nor RFD was affected in either muscle [71]. However, more recent evidence using a fatiguing, mechanically controlled stretch-shortening work cycle model has shown that NaHCO_3_ administration may improve RFD [83]. Avoiding the limited degree of shortening that occurs during isometric contractions [71], Higgins and colleagues subjected both SOL and EDL mouse muscles to repeated length changes matched to the expected stride frequency of the mice during normal locomotion (10 % strain with a cycle frequency for EDL of 8 Hz and SOL of 5 Hz) and demonstrated an approximate 7 % improvement in force production during shortening in the EDL compared to a 3 % improvement in the SOL after NaHCO_3_ incubation.

To date, the fibre-type dependent findings related to contractile properties have not been replicated in the exercising human given the methodological constraints addressed earlier of isolating fibre-type contributions to whole-body locomotion [84], quantifying properties of diffusion and other factors associated with intramuscular blood supply [85, 86]. However, collectively, this body of research suggests that NaHCO_3_ may affect the metabolic properties associated with contractile shortening velocity and that this effect appears more pronounced in fast-twitch fibres (Fig. 2). The effect on rates of contractile shortening but not maximal force may also explain the limited efficacy of NaHCO_3_ observed in performance-based studies only measuring maximal voluntary force [87, 88].

#### Chronic Adaptation

Interestingly, although shortening velocity of slow-twitch fibres after acute NaHCO_3_ administration does not appear to be improved when compared to fast-twitch fibres, chronic ingestion coupled with high-intensity training may improve the oxidative capacity of these fibres [89–91]. After 8 weeks (three times per week) of chronic NaHCO_3_ ingestion coupled with high-intensity (between 140 and 170 % lactate threshold (LT)) intermittent training, Edge and colleagues reported a significant improvement in both LT (~11 %) and performance (~25 %) (time to failure) in moderately trained females when compared to a control group matched for total work (kJ). The authors speculated that as H^+^ accumulation has been shown to impair oxidative capacity and various components of cellular respiration [57], training in an alkalotic state may have attenuated the impairment and allowed for prolonged cellular respiration to occur during training [89].

Continuing this line of investigation, two subsequent studies by the same group used relatively similar NaHCO_3_ dosing and high-intensity training protocols on rats to explore fibre-type dependent protein expression associated with H^+^ removal during rest and exercise [90, 91]. In the first study, the authors observed a differential effect of alkalosis on MCT-4 expression in the SOL but not EDL when compared to placebo and control conditions [90]. Additionally, citrate synthase concentration was higher in the alkalotic condition (23 vs. 16 %) after training in the SOL but not EDL. These findings led the authors to conclude that chronic NaHCO_3_ ingestion was influencing protein expression associated with slow but not fast-twitch fibres. The final study in the series appeared to further confirm this observation, with the authors reporting an increase in mitochondrial respiration in the SOL as compared to the EDL after training in an induced alkalotic state [91].

The evidence that chronic use of NaHCO_3_ coupled with appropriate training may lead to aerobic adaptations associated with improved mitochondrial efficiency in slow-twitch fibres is intriguing [91], and future work may clarify whether this is indeed a viable option for improving aerobic efficiency. Additionally, we are unaware of any studies that have investigated the efficacy of introducing chronic NaHCO_3_ supplementation on mechanisms associated with muscle force production or rapid force-generating capacity. Given the preliminary evidence that NaHCO_3_ may exhibit independent effects on fibre-specific mechanisms associated with improved contractile function both acutely and chronically, future study in this area is warranted.

### Motor Pathways

Ultimately, determining the mechanisms by which NaHCO_3_ supplementation can improve athletic performance may require a more integrative understanding of other systems inherently involved during exercise, and more specifically the progression of skeletal muscle fatigue. The motor pathways, or more explicitly the neuromuscular system, are intimately involved in maintaining skeletal muscle function during fatiguing exercise. This complex integrative network is often overlooked in the NaHCO_3_ literature as a possible contributor to whole-body exercise performance. Although partially due to the limited number of studies observing the influence of NaHCO_3_ on the motor pathways, studies in this section have been broadly categorised as they relate to motor unit recruitment and feedback mechanisms associated with metabolite accumulation in the periphery that have been directly linked to central drive.

As addressed in the ‘Whole-body Metabolism’ section, fluctuations in strong ions (e.g. K^+^, Na^+^, Cl^−^) have also been linked with maintaining resting membrane potential and sarcolemmal excitability [13]. However, few studies have explored whether inducing pre-exercise alkalosis via NaHCO_3_ ingestion influences these mechanisms as they relate to excitation-contraction coupling and ultimately muscle force generating capacity [92–95]. Hunter and colleagues investigated the effects of NaHCO_3_ ingestion on muscle fibre conduction velocity after fatiguing exercise (i.e. 50 min of cycling at 105 % lactate threshold) [93]. Using a previously developed technique [96], these authors observed an attenuation in the decline in muscle fibre conduction velocity after cycling in an alkalotic state [93]. This improvement, however, did not translate into an improvement in maximal knee extension force.

We have also demonstrated that NaHCO_3_ has no effect on maximal force production [8]. However, we have observed an improvement in the ability to rapidly generate force (RFD), a functional measure related to both rapid motor unit recruitment and contractile shortening velocity [97]. In this study, subjects performed an intermittent (30 s:30 s work-to-rest ratio) cycling protocol at 120 % of peak power until volitional exhaustion, where maximal voluntary contractions (MVC) were performed immediately after each 30-s work cycle. Although RFD and maximal force declined in both NaHCO_3_ and control trials, RFD was better maintained throughout the exercise under alkalotic conditions [97]. The mechanisms responsible for the differential effect on RFD but not maximal force after NaHCO_3_ ingestion requires further investigation, as RFD is influenced by both peripheral (e.g. excitation-contraction coupling) and central (e.g. central drive) factors [98].

Mechanisms related to central drive such as cortical output, spinal reflexes and muscle afferents have also been shown to be influenced by the localised changes in muscle metabolite concentrations (e.g. H^+^) associated with exercise [99–101]. The complex interaction between these mechanisms as they contribute to skeletal muscle fatigue has been previously reviewed [99]. Briefly, they are often categorised as supraspinal (e.g. output of descending motor cortical and corticospinal pathways to the motoneurons) and sub-spinal (e.g. muscle spindles, Golgi tendon organs, small diameter group III/IV afferents) mechanisms [99] and their relative contribution to skeletal muscle fatigue associated with the intensity of the exercise task. The degree to which H^+^ accumulation in the periphery affects central mechanisms, particularly during intense exercise, has been related specifically to the central projection of the mechanically and chemically sensitive small diameter group III/IV afferents located within the skeletal muscle [102, 103]. It is believed that the exercise-induced actions of these afferent fibres inhibit the excitability and net output of corticospinal cells in the central nervous system, as well as effect alpha motor neuron excitability [104–107].

Very few investigations have used NaHCO_3_, or indeed other methods, to manipulate pH and to explore the effects of alkalosis on afferent or other neural pathways contributing to skeletal muscle fatigue [94, 95]. Based on the assumption that H^+^ accumulation leads to a decline in central drive to the muscle, Matsuura and colleagues measured surface electromyography (sEMG) activity in the vastus lateralis to determine whether motor unit recruitment strategies during repeated high-intensity cycling (i.e. repeated 10-s efforts at a resistive load (*N*) of 0.075 BM 9.81^−1^) would be influenced by NaHCO_3_ ingestion [94]. Neither root mean square or mean power frequency was influenced by pH, with both NaHCO_3_ and control conditions reflecting similar recruitment profiles throughout the test [94]. A follow-up study by the same group suggested that NaHCO_3_ again had no effect on gross sEMG activity or perceived sense of effort after 2 min of cycling at 105–110 % of a pre-determined maximal workload [95]. However, sEMG signals lack sensitivity to detect changes in the number and discharge rate of active motor units that would indicate increased central motor output [108, 109] and therefore may not be sensitive enough to accurately determine whether or not altering pH may affect descending central drive.

A more recent body of literature suggests a relationship between increasing levels of intramuscular metabolite concentrations analogous with high rates of skeletal muscle contraction, and greater group III/IV afferent firing in humans [110–112], which would ultimately act as a protective mechanism against peripheral fatigue by limiting descending central drive [113]. Using an isometric knee extension model in combination with ischemia to enhance the firing frequency of group III/IV afferents [114–116], we have observed voluntary activation, a gross estimate of central drive, to be better preserved under NaHCO_3_ conditions [117]. The improved activation during alkalosis, however, was not commensurate with changes in net muscle excitation or rapid and maximal force output which declined equally in both NaHCO_3_ and control conditions [117]. We speculated that the divergence might have been a result of the extreme conditions of peripheral fatigue induced by our exercise protocol (2-min MVC of the quadriceps followed by 2 min of ischemia and ending with a 1-min ischemic MVC) [117]. The findings may also reflect the relatively minor role afferent feedback may play in these situations of extreme peripheral fatigue, where other factors separate to group III and IV afferents may have contributed to the down-regulation of descending central drive.

The use of stimulation (e.g. nerve, muscle, transcranial magnetic stimulation) to explore the potential link between NaHCO_3_ and central and peripheral mechanisms associated with skeletal muscle fatigue may provide further insight toward the ergogenic potential of this supplement. Moreover, our findings that alkalosis may affect central mechanisms associated with early rate of torque development warrants further investigation, as the ability to rapidly recruit muscle may be influenced by the contractile properties of the fibre (e.g. type I or II) (Fig. 2) [118]. As evidence suggests that acute NaHCO_3_ ingestion may have a preferential effect on type II muscle fibres [83], further study comparing different combinations of exercise task demands and muscle groups may also clarify the effect of NaHCO_3_ on rates of torque development and muscle recruitment in humans.

### Performance Applications

#### Competition and Training Recommendations

Appropriately identifying whether NaHCO_3_ ingestion may influence the underlying mechanisms associated with whole-body metabolism, muscle physiology and/or motor pathways within the context of a particular athletic endeavour or training stimulus is an important initial step when considering the use of this supplement. For example, in the context of athletic performance, delayed onset of H^+^ accumulation may be relevant to certain time frames within an event that demand rapid transitions between steady-state and higher intensity efforts (e.g. beginning a ‘long-finish’ earlier in a 1500-m race). In this scenario, NaHCO_3_ supplementation may be appropriate based on the evidence indicating an attenuation of H^+^ accumulation during exercise intensities transitioning between aerobic and anaerobic pathways [60, 65, 89]. Another example, and specifically related to the evidence surrounding RFD and fast-twitch fibres [83, 97], would be to improve the propulsive force at the start of an explosive movement (e.g. the initiation of a pedal stroke). Considerations for the potential benefit of NaHCO_3_ in these unique situations would require extensive consultation within an athlete’s support network, as well as a systematic integration of the supplement to accurately determine any efficacious response. Although often difficult to rigorously assess in the elite environment, any marginal improvement in performance gained by using NaHCO_3_ for these specific purposes in competition may provide alternative uses for this supplement that extend beyond current recommendations [1, 5].

Although there have been few studies investigating the efficacy of implementing a chronic NaHCO_3_ supplementation strategy on training outcomes [89, 119], given the recent evidence in animal models exhibiting enhanced oxidative/mitochondrial adaptations in slow-twitch fibres after chronic supplementation [90, 91], further work in this area may establish whether incorporating NaHCO_3_ into an aerobic training paradigm is warranted. Furthermore, with research also supporting a potential ergogenic effect on RFD [97], particularly in fast-twitch fibres [83], applying a loading strategy during specific training and adaptation blocks (e.g. explosive power) may also have merit. In practice, however, incorporating a training strategy inclusive of a regular 0.3 g·kg^−1^ NaHCO_3_ load would require a systematic administration and monitored approach. Although we are unaware of any documented long-term adverse effects of chronic loading in either sporting [89, 120, 121] or clinical contexts [122–124], given the relatively high sodium content (e.g. a 70-kg athlete would consume ~6 g), continually monitoring both blood acid-base and strong ions (e.g. Na^+^) would allow any irregularities from the ingestion regimen to be detected and ultimately corrected for the safety of the athlete(s). Additionally, and similar to other contemporary debates such as training in a low glycogen state [125], without further study potentially elucidating any maladaptation from consecutive training blocks after NaHCO_3_ ingestion, it would be prudent to consider intermittently incorporating this strategy and only when certain training outcomes are desired (e.g. maximising the number of repeated efforts at top speed or high rates of force output).

#### Loading Recommendations

Equally important to the performance and training strategies, however, may be the ingestion protocol used to systematically introduce NaHCO_3_. Research is only now highlighting what practitioners, coaches and athletes have been aware of for some time, and that is the inherent variability in individual responsiveness to this supplement [126–130]. In part, the variability in many papers may eventuate from the wide range of exercise tasks and methodological applications, as well as heterogeneity between participant phenotypes. However, it may also result from the widespread assumption that applying mean data on time-to-peak buffering response rates after NaHCO_3_ ingestion prior to commencing exercise is not influencing the outcome of many performance tasks [128, 129]. Amongst others [131, 132], we have profiled the ingestion time course of various doses of NaHCO_3_ at rest in order to determine peak response rates (Fig. 1) [3]. Collectively, and variation in ingestion protocols notwithstanding (e.g. capsules, bolus loads or dispersed over time), these studies illustrate around 40 min of variation between subjects’ peak in blood buffering capacity [3, 131, 132]. Indeed, the large degree of inter-individual variability profiled in Fig. 1 has been recently observed again but under greater temporal resolution and in a larger study cohort [129]. Although we have shown that this variation may not be important for recreationally trained individuals [133], we are unaware of any studies that have documented the variability in elite athlete populations.

Finally, the origin of the 0.2 to 0.3 g·kg^−1^ doses in the sport performance field is somewhat ambiguous. Although oral ingestion of between 25 to 40 g of NaHCO_3_ has been documented as early as the 1920s and early 1930s in the clinical literature [124, 134, 135], this range is believed to have come from the work of Poulus and colleagues, who in 1974 published the first exercise performance paper using a physiochemical rationale for NaHCO_3_ dose selection [74]. Citing earlier clinical work [122, 136], these authors determined the amount of NaHCO_3_ to be administered intravenously using the following formula:

$$ {\mathrm{NaHCO}}_3 = \mathrm{subject}\ \mathrm{body}\ \mathrm{weight}\ \left(\mathrm{kg}\right) \times 0.3 \times \mathrm{base}\ \mathrm{deficit} $$

where base deficit (BD) represented the excess of fixed acid in the blood measured after an incremental cycling test on a previous visit to the lab and compared to a standard (pH of 7.4 and a PCO_2_ of 40 mmHg) [74]. The 0.3 represented a mean weighting factor derived from the equilibrating time course of NaHCO_3_ throughout blood and the extravascular spaces [122, 123]. Of note, no subsequent studies have included BD in the equation for determining dose. This, coupled with the variation in individual weighting factors reported in the original study (0.25 to 0.38) [122], warrants further work to determine whether or not individualising dose concentrations is necessary, and whether issues related to HCO_3_^−^ and [H^+^] rates of increase and clearance are impacting upon the efficacy of NaHCO_3_ as a supplement [130].

The dosing papers most frequently cited in the sport performance literature date back to the late 1980s and early 1990s. These studies looked at a range of NaHCO_3_ doses (0.1 to 0.5 g·kg^−1^) and the relationship between performance and gastrointestinal (GI) disturbance, respectively [137, 138]. Collectively, these studies showed that performance could be improved when the acute dose ingested ranged between 0.2 and 0.3 g·kg^−1^, but anything greater was not beneficial and only induced severe side effects. For the past 30 years, this dose range has been widely accepted as best practice. Yet, the small subject numbers (*n* < 9) coupled with their non-elite training status (e.g. ‘participating in athletic events’) of the original research should warrant further inquiry [138]. This, of course, also assumes that 0.2 or 0.3 g·kg^−1^ are the optimal physiologic doses for maximising blood buffering capacity [130].

In light of the practical issues identified in this section, we recommend monitoring at an individual level the time-to-peak rise (and decay [130]) in [HCO_3_^−^] at doses between 0.2 and 0.3 g·kg^−1^ and to tailor the commencement of exercise accordingly [128]. An individualised loading strategy will also require systematically incorporating dose distribution and other planned nutritional interventions, documenting subjective feedback from the athlete (e.g. gastrointestinal distress [139]) and monitoring any physiological changes (e.g. acute weight gain from plasma volume expansion [21]) observed after ingestion.

## Conclusions

We have attempted to provide a comprehensive overview of the mechanistic links between skeletal muscle fatigue, metabolic acidosis and NaHCO_3_ supplementation. The synergistic associations presented within the ‘Whole-body Metabolism’, ‘Muscle Physiology’ and ‘Motor Pathways’ sections provide evidence that an integrative perspective is required to truly understand and optimise the use and application of this supplement. By consolidating the mechanistic evidence into one text, we aimed to provide new insights and possibly direct future research in the field of NaHCO_3_ supplementation. Finally, we hope that this review provides further evidence base to expand and refine the use of this supplement to improve athletic performance beyond traditional methods of application.
